# High Dose Intravenous Vitamin C for Preventing The Disease Aggravation of Moderate COVID-19 Pneumonia. A Retrospective Propensity Matched Before-After Study

**DOI:** 10.3389/fphar.2021.638556

**Published:** 2021-04-22

**Authors:** Bing Zhao, Min Liu, Ping Liu, Yibing Peng, Jun Huang, Mengjiao Li, Yihui Wang, LiLi Xu, Silei Sun, Xing Qi, Yun Ling, Jian Li, Wenhong Zhang, Enqiang Mao, Jieming Qu

**Affiliations:** ^1^ Department of Emergency of Ruijin Hospital, Shanghai Jiao Tong University School of Medicine, Shanghai, China; ^2^ Department of Gastroenterology, Shanghai Public Health Clinical Center, Shanghai, China; ^3^ Department of Tuberculosis, Shanghai Public Health Clinical Center, Fudan University, Shanghai, China; ^4^ Department of Laboratory Medicine, Ruijin Hospital, School of Medicine Shanghai Jiaotong University, Shanghai, China; ^5^ Shanghai Institute of Hypertension, Shanghai, China; ^6^ Department of Infectious Disease, Shanghai Public Health Clinical Center, Shanghai, China; ^7^ Clinical Research Center in Ruijin Hospital, School of Medicine Shanghai Jiao Tong University, Shanghai, China; ^8^ Department of Infectious Disease of Shanghai Huashan Hospital, Fudan University, Shanghai, China; ^9^ Department of Respiratory and Critical Care Medicine of Ruijin Hospital, Shanghai Jiao Tong University School of Medicine, Shanghai, China

**Keywords:** COVID-19, vitamin C, therapy, inflammatory response, disease aggravation

## Abstract

**Background:** Coronavirus disease 2019 (COVID-19) pandemic is continuing to impact multiple countries worldwide and effective treatment options are still being developed. In this study, we investigate the potential of high-dose intravenous vitamin C (HDIVC) in the prevention of moderate COVID-19 disease aggravation.

**Methods:** In this retrospective before-after case-matched clinical study, we compare the outcome and clinical courses of patients with moderate COVID-19 patients who were treated with an HDIVC protocol (intravenous injection of vitamin C, 100 mg/kg/day, 1 g/h, for 7 days from admission) during a one-month period (between March 18 and april 18, 2020, HDIVC group) with a control group treated without the HDIVC protocol during the preceding two months (January 18 to March 18, 2020). Patients in the two groups were matched in a 1:1 ratio according to age and gender.

**Results:** The HDIVC and control groups each comprised 55 patients. For the primary outcomes, there was a significant difference in the number of patients that evolved from moderate to severe type between the two groups (HDIVC: 4/55 vs. control: 12/55, relative risk [RR] = 0.28 [0.08, 0.93], *P* = 0.03). Compared to the control group, there was a shorter duration of systemic inflammatory response syndrome (SIRS) (*P* = 0.0004) during the first week and lower SIRS occurrence (2/21 vs 10/22, *P* = 0.0086) on Day 7 (6–7 days after admission). In addition, HDIVC group had lower C-reactive protein levels (*P* = 0.005) and higher number of CD4^+^ T cells from Day 0 (on admission) to Day 7 (*P* = 0.04).” The levels of coagulation indicators, including activated partial thromboplastin time and D-dimer were also improved in the HDIVC compared to the control group on Day 7.

**Conclusion:** HDIVC may be beneficial in limiting disease aggravation in the early stage of COVID-19 pneumonia, which may be related to its improvements on the inflammatory response, immune function and coagulation function. Further randomized controlled trials are required to augment these findings.

## Introduction

The potentially fatal disease, coronavirus disease 2019 (COVID-19), has caused a worldwide pandemic since December 2019 (Mahase, 2020; Spinelli and Pellino, 2020). By September 10, 2020, SARS-CoV-2 had affected more than 200 countries, resulting in more than 28 million confirmed cases, and over 900,000 confirmed deaths. Besides Corticosteroids for severe and critical COVID-19, few agents have been shown to be definitively effective according to the latest guideline of World Health Organization (Anonymous, 2020). By severity, COVID-19 is classified into mild, moderate, severe, and critical type according to the guidelines of the National Health and Family Planning Commission of the People’s Republic of China (National Health and Family Planning Commission of the People’s Republic of China, 2020).

The severe type is mainly characterized by deteriorating respiratory function and rapid progression of radiological lesions, while the critical type further requires mechanical ventilation and is accompanied by shock or multiple organ failure. These two types are reported to be associated with a mortality rate as high as 66% (Wu et al., 2020). One of the keys to improving the prognosis of COVID-19 is to prevent disease aggravation, especially when the disease severity ranges from moderate, through severe, to critical type.

High dose intravenous vitamin C (HDIVC) has been suggested to exert beneficial effects on various critical illnesses in animal and clinical studies (Oudemans-van Straaten et al., 2014). HDIVC was shown to reduce 28-days all-cause mortality (29.8 vs 46.3%, *P* = 0.01) by sepsis in the CITRIS-ALI study (Fowler et al., 2019), and this result was recently reanalyzed by Hemilä and Chalker (2020) who revealed stronger evidence when the analysis is restricted to the four days during which vitamin C was administered (mortality, 4.8 vs 22.9%, *P* = 0.0007). Conversely, another recent trial, ACT, found that a combination of vitamin C, corticosteroid and thiamine exerted no beneficial effect on organ function (Moskowitz et al., 2020). The rationale for HDIVC administration in the treatment of COVID-19 patients, as we speculated, relies on its ability to effectively eliminate the surge of reactive oxygen species and the ensuing uncontrolled inflammatory response and organ dysfunction. Additionally, vitamin C has been demonstrated to have potential immune-enhancing properties, which may help to improve lymphopenia, the main characteristic of COVID-19 that is associated with severity (Wang et al., 2020). The administration of HDIVC in COVID-19 has already received much attention (Carr and Rowe, 2020; Cerullo et al., 2020). In this retrospective before-after case-matched study, we investigate whether HDIVC could prevent disease aggravation from the moderate to the severe type and its effect on the inflammatory response, immune function, and organ function.

## Methods

### Study Design and Participants

**FIGURE 1:**
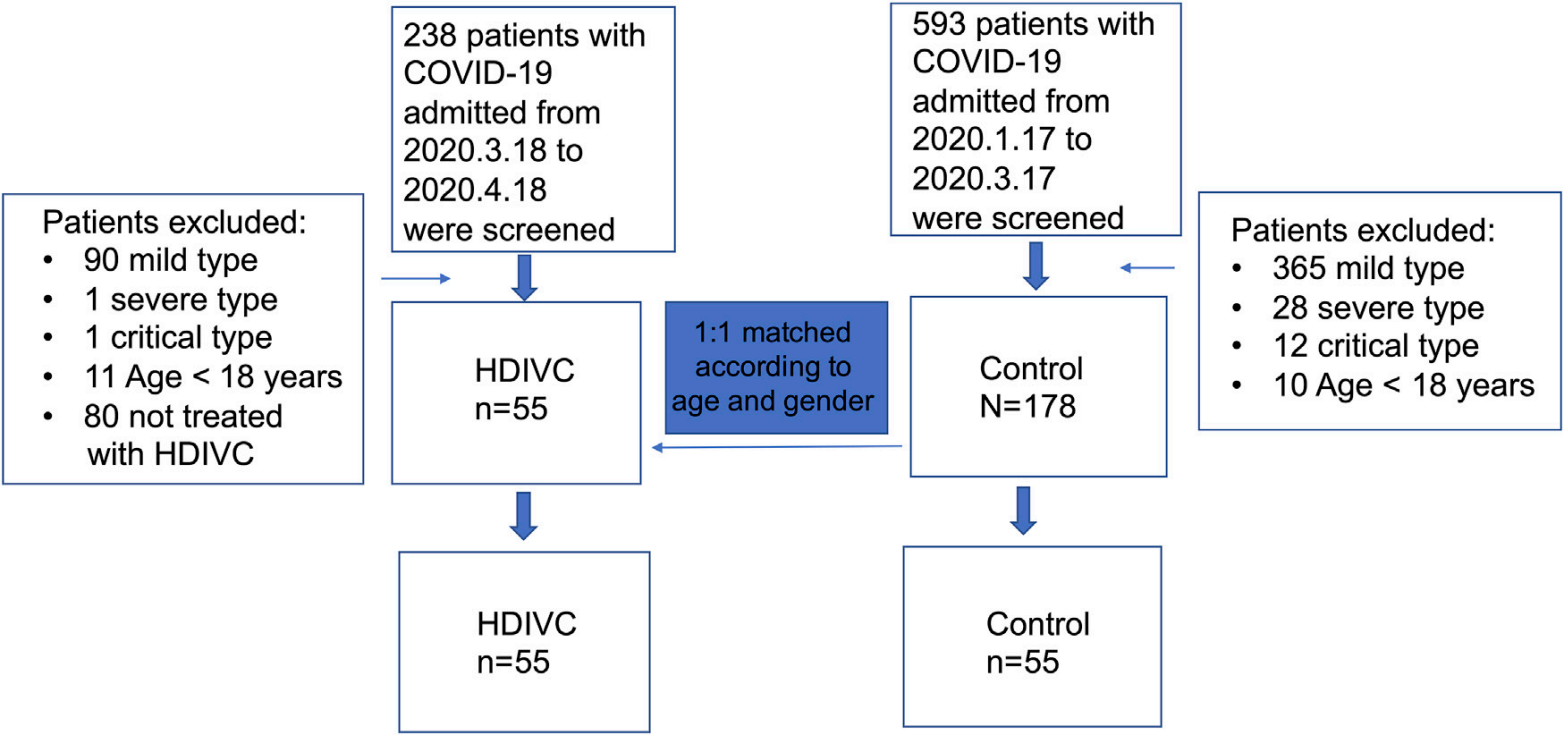
Study flowchart. HDIVC, high dose intravenous vitamin C; COVID-19, coronavirus disease 2019.

This study was an electronic health record-based retrospective before-after case-matched clinical study. It was conducted in accordance with the amended Declaration of Helsinki (as revised in 2013) and approved by the Institutional Ethics Board of the Ruijin Hospital, Shanghai Jiao Tong University school of medicine, and has been retrospectively registered in the Chinese Clinical Trail Registry (ChiCTR2000033050). This study was conducted at the Shanghai Public Health Clinical Center. From March 18, 2020, we began to use the HDIVC protocol in the treatment of COVID-19 patients. To investigate the effect of HDIVC in the prevention of disease aggravation, we screened the patients admitted between March 18, 2020 and april 18, 2020 who accepted HDIVC treatment. The inclusion criteria for the HDIVC group were:

1) COVID-19 patients with a diagnosis of moderate type on admission; 2) age >18 years; 3) patients who were not pregnant and had no malignant tumors. The diagnosis and severity classification followed the guidelines of the National Health and Family Planning Commission of the People’s Republic of China (National Health and Family Planning Commission of the People’s Republic of China, 2020). For the control group, we retrospectively screened the patients who had been admitted during the two previous months (between January 17, 2020 and March 17, 2020) according to the same criteria as those in the HDIVC group. These patients had not received the HDIVC protocol Propensity score matching was conducted to minimize the impact of potential confounders and selection bias between 2 groups of patients. A propensity score for each patient was calculated through logistic regression modeling and covariates of age and gender were matched. A 1:1 matching was used to select patients in the 2 groups, with the caliper width set as 0.1 for the standard deviation (Figure 1).

### Data Collection

Data were collected from an electronic medical records and reviewed by two trained physicians. The observation period was the first week after admission. The information or data were collected mainly on admission (“Day 0”), 3–4 days (“Day 3”), and 6–7 days (“Day 7”) after admission. Information regarding age, gender, body weight, co-existing diseases, and epidemiology was obtained. The definition of systemic inflammatory response syndrome (SIRS) has been described previously (Kaukonen et al., 2015). Data regarding the serum levels of C-reactive protein (CRP), erythrocyte sedimentation rate (ESR), and the occurrence and duration of SIRS were also collected. Additionally, data regarding immune indicators, including counts of CD4^+^ T cells, CD8^+^ T cells, and lymphocytes were collected. Indicators of organ function, including lactate dehydrogenase (LDH), total bilirubin (TB), alanine transaminase (ALT), activated partial thromboplastin time (APTT), creatine kinase (CK), cardiac troponin I (cTNI), and pre-albumin levels were also recorded.

The primary outcome was disease aggravation, defined as a progression of the disease severity from moderate type on admission to severe type within one week after admission. The clinical symptoms of the mild type are non-severe, with no pneumonia on imaging examination. The moderate type is characterized by symptoms and pneumonia-related imaging findings. The severe type is diagnosed if any of the following criteria was met: 1) respiratory rate ≥30 cycles/minute; 2) in the resting state, arterial oxygen saturation (SaO2) ≤93%; arterial partial pressure of oxygen/fraction of inspired oxygen ≤300 mmHg; 3) pulmonary imaging shows lesions that have progressed by more than 50% within 24–48 h. The critical type is diagnosed if any of the followings criteria was met: 1) patient require mechanical ventilation; 2) shock occurs; 3) combination with other organ failure that requires ICU monitoring and treatment. The secondary outcomes included indicators for inflammatory response, immune function, organ function and time to viral load negative (Supplementary Table S1).

### Treatment Protocol

All patients received treatment based on the guidelines of the National Health and Family Planning Commission of the People’s Republic of China (National Health and Family Planning Commission of the People’s Republic of China, 2020) and the Shanghai expert consensus on comprehensive treatment of COVID-19 (Shanghai Expert Group on Clinical Treatment of New Coronavirus Diseases, 2020). The HDIVC protocol for moderate COVID-19 consisted of an intravenous injection of vitamin C (ascorbic acid) at a dosage of 100 mg/kg/day and a rate of 1 g/h for 7 days, starting from the time of admission. Other associated therapies included antiviral therapy, nutrition support, the low-molecular-weight heparin (if D-dimer was above the normal value), antibiotics in cases of suspected bacterial infections, nasal tube oxygen support if necessary, and/or physical cooling and medical treatment (non-steroidal anti-inflammatory drugs or glucocorticoid) if the body temperature was above 38°C.

### Statistical Analysis

Continuous variables were presented as medians and interquartile range (IQR, shown in square brackets) and compared using the Mann-Whitney U test, or reported as the mean with standard deviation and compared using the *t*-test as per distribution type. Categorical variables were compared using Fisher’s exact test. The generalized estimating equations (GEE) were performed to investigate the difference in inflammatory markers, immune function, and organ function between the HDIVC and control groups. All statistical analyses were performed using SAS v. 9.2 (SAS Institute Inc., United States) and GraphPad prism 8.0 (version 8.2.0). Two-sided P values of less than 0.05 were considered statistically significant.

## Results

### Characteristics of the Patients

As Figure 1 shows, 238 patients, admitted between March 18, 2020 and april 18, 2020, were retrospectively screened, and 55 patients met the inclusion criteria for the HDIVC group. Between January 17, 2020 and March 17, 2020, 593 patients admitted to the Shanghai Clinic Public Health Center were screened for the purpose of matching. One hundred and seventy-eight patients diagnosed with moderate COVID-19 on admission were selected to match patients in the HDIVC group in a 1:1 ratio according to age and gender. Fifty-five patients were included in the control group. Patient characteristics were similar between the HDIVC and control groups (Table 1). The main associated therapies within the first weeks after admission included antiviral therapy, antibiotics, low-molecular-weight heparin, and glucocorticoids. No significant difference in therapies was found between the two groups.

**TABLE 1:** Characteristics of COVID-19 patients.

	HDIVC (*n* = 55)	Control (*n* = 55)	*P* Value
**Age, median (IQR), y**	36 (31–47)	36 (31–46)	0.96
**Sex (male, n)**	33	35	0.69
**Weight, median (IQR), kg**	70 (58–80)	65 (55–76)	0.26
**Interval from first symptom to admission, median (IQR), days**	4 (2–6)	3 (2–7)	0.65
**Symptoms on Day 0**			
Fever (n)	34	43	0.06
Dry cough (n)	27	32	0.33
Diarrhea (n)	6	4	0.51
Olfactory dysfunction (n)	3	0	0.07
Gustatory dysfunction (n)	2	0	0.15
**Co-existing disease**			
Hypertension (n)	1	6	0.05
Diabetes (n)	3	4	0.69
**Contemporary treatments, n**			
Antiviral (n)	52	54	0.31
Antibiotic (n)	12	20	0.06
Low molecular heparin (n)	16	10	0.44
Glucocorticoid (n)	2	5	0.24

HDIVC, high dose intravenous vitamin C; IQR, interquartile range; Day 0, the day on admission. P, HDIVC vs control group.

### Effect of High Dose Intravenous Vitamin C on Primary Outcome

**FIGURE 2:**
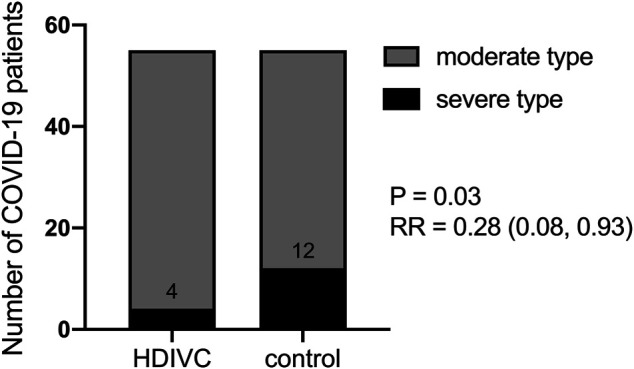
Effect of HDIVC on preventing the disease aggravation. The number of patients who experienced disease aggravation in HDIVC and the control group were compared (4/55 vs 12/55, RR = 0.28 [0.08, 093], *P* = 0.03). HDIVC, high dose intravenous vitamin C; COVID-19, coronavirus disease 2019; RR, relative risk; CI, confidential interval.

The primary outcome is to investigate if HDIVC could prevent disease aggravation. All enrolled patients were diagnosed with moderate COVID-19 on admission (Day 0). As Figure 2 showed, at the end of the observational period (Day 7), 4 patients in the HDIVC group and 12 in the control group suffered the disease aggravation with a final diagnosis of severe or critical COVID-19 (relative risk [RR] 95% confidential interval [CI] = 0.28 [0.08, 0.93], *P* = 0.03). IQR is shown in square brackets.

### Effect of High Dose Intravenous Vitamin C on Secondary Outcomes

As Table 2 shows, SIRS occurrence at Day 0 was similar between the two groups (HDIVC: 21/55vs. control: 22/55; RR = 0.93 [0.43–1.93], *P* = 0.86). On Day 7, there were fewer patients with SIRS in the HDIVC group (*N* = 2/21) than the control group (*N* = 10/22, RR = 0.13 [0.02–0.68], *P* = 0.0086). Among the patients with SIRS on admission, the duration of SIRS was further analyzed, and we found that patients who accepted the HDIVC protocol experienced a significantly shorter lasting time of SIRS (2 [1, 3], days) than the ones who did not (6 [1, 7], days, *P* = 0.0004). There was no significant difference in the serum levels of CRP between the HDIVC group and the control group on Day 0 and Day 3. However, on Day 7, CRP levels were significantly lower in the HDIVC group than in the control group (0.5 [0.5, 0.6] vs 0.5 [0.5, 7.7], mg/L, *P* = 0.005). Another inflammatory indicator, ESR, showed no significant difference between the two groups.

**TABLE 2:** Effect of HDIVC on inflammatory response.

Variables	Time points	n	HDIVC	N	Control	RR (95%CI)	P Value
**Patients with SIRS, n/total**	Day 0	55	21/55	55	22/55	0.93 (0.43–1.93)	0.85
**Patients with SIRS, n/total**	Day 7	21	2/21	22	10/22	0.13 (0.02–0.68)	**0.008**
**Duration of SIRS, days, median (IQR)**	Day 0 to day 7	55	2 (1, 3)	55	6 (1, 7)	—	**0.0006**
**Serum level of CRP**	Day 0	55	1.2 (0.5, 7.6)	55	0.5 (0.5, 7.3)	—	0.19
**mg/L, median (IQR)**	Day 3	55	0.5 (0.5, 8.5)	55	0.5 (0.5, 10.2)	—	0.18
	Day 7	55	0.5 (0.5, 0.6)	54	0.5 (0.5, 7.7)	—	**0.02**
**Serum level of ESR**	Day 0	55	33 (10, 76)	50	40.5 (21, 74.3)	—	0.23
**ml/h, median (IQR)**	Day 3	45	44 (21, 75)	49	39 (23.5, 72)	—	0.39
	Day 7	48	30 (11, 49.8)	47	38 (21, 73)	—	0.09

HDIVC, high dose intravenous vitamin C; RR, relative risk; CI, confidential interval. SIRS, systemic inflammatory response syndrome; CRP, C-reactive protein; ESR, erythrocyte sedimentation rate; IQR, interquartile range; Day 0, the day on admission; Day 3, 3–4 days after admission; Day 7, 6–7 days after admission. P, HDIVC vs control group.

As Table 3 shows, for the patients with CD4^+^ T lymphocyte deficiency (<410/μL) on admission, HDIVC exerted a significant improving effect (334 [191.9, 409.3] vs 151 [43.5, 240] *P* = 0.04), but not for the patients with deficiencies in CD8^+^ (190/μL) and lymphocytes on admission. There was no obvious effect of HDIVC on the CD4^+^ T cell counts, CD8^+^ T cell counts, and lymphocytes counts on Day 3 and Day 7 for the entire study population (Supplementary Table S2).

**TABLE 3:** Effect of HDIVC on the recovery of immune function deficiency.

Variables (median [IQR])	Time points	n	HDIVC	n	Control	RR (CI)	P Value
**Patients with CD4** ^**+**^ **T cell (<410/μL) deficiency on day 0, n/total**	Day 0	12	12/55	18	18/55	0.6 (0.2–1.3)	0.19
Counts of CD4^+^ T cell, n/μl	Day 0	12	289.5 (262.3, 339.3)	18	340 (203, 375)	—	0.29
Counts of CD4^+^ T cell, n/μl	Day 7	12	638 (452.3, 746.5)	9	493 (281.5, 641.5)	—	0.17
Increase of CD4^+^ T cell, n/μl	Day 0 to day 7	12	334 (191.9, 409.3)	9	151 (43.5, 240)	—	**0.04**
**Patients with CD8** ^**+**^ **T cell deficiency (<190/μL) on day 0, n/total**	Day 0	4	4/55	9	9/55	0.4 (0.1–1.4)	0.14
Counts of CD8^+^ cell, n/μl	Day 0	4	143 (95.5, 163.5)	9	125 (108, 166)	—	>0.9
Counts of CD8^+^ cell, n/μl	Day 7	4	240 (215.5, 346.3)	6	287 (147, 339.5)	—	>0.9
Increase of CD8^+^ T cell, n/μl	Day 0 to Day 7	4	123 (65, 211.8)	6	153 (51.5, 242.9)	—	0.76
Patients with lymphocyte deficiency (<1.1 *10^9/L) on Day 0, n/total, %	Day 0	13	13/55, 23.6	19	19/55, 34.5	0.6 (0.3–1.4)	0.21
Counts of lymphocyte, n*10^9/L	Day 0	13	0.9 (0.7, 1.1)	19	0.8 (0.7, 1)	—	0.22
Counts of lymphocyte, n*10^9/L	Day 7	13	1.4 (1.2, 1.9)	18	1.2 (0.7, 1.6)	—	0.11
Increase of lymphocyte, n*10^9/L	Day 0 to Day 7	13	0.5 (0.4, 1.1)	18	0.35 (-0.02, 0.76)	—	0.09

The COVID-19 patients with a deficiency of CD4^+^ T cells, CD8^+^ T cells and lymphocytes on Day 0 were selected. The increases in these immune cells from Day 0 to Day 7 were compared between the HDIVC and control group. IQR, interquartile range; RR, relative risk; CI, confidential interval; HDIVC, high dose intravenous vitamin C; Day 0, the day on admission; Day 3, 3–4 days after admission; Day 7, 6–7 days after admission. P, HDIVC vs control group.

As Table 4 shows, D-dimer levels in the HDIVC group (0.3 [0.2, 0.4], μg/ml) were lower than those in the control group (0.4 [0.2, 0.7], μg/ml, P = 0.05). APTT in the HDIVC group (seconds) was significantly shorter than that in the control group on Day 3 (37.7 [35.2, 39.3] vs 40.1 [36.8, 44.2], seconds, *P* = 0.02) and Day 7 (36.9 (34.9, 38.9) vs 40.8 (36.5, 43.5), seconds, *P* = 0.02). Other organ function indicators including LDH, TB, ALT, D-Dimer, APTT, cTNI, and CK-MB were within the normal ranges on Day 0 and showed no obvious changes in either of the two groups on Day 3 and Day 7.

**TABLE 4:** Effect of HDIVC on organ functions.

Variables	Time points	HDIVC		Control		*P* Value
		n	Median (IQR)	n	Median (IQR)	
**DD (μg/ml)**	Day 0	55	0.3 (0.2, 0.5)	55	0.3 (0.2, 0.4)	0.84
**(0–0.5)**	Day 3	45	0.4 (0.3, 0.5)	50	0.3 (0.2, 0.4)	0.80
	Day 7	51	0.3 (0.2, 0.4)	52	0.4 (0.2, 0.7)	**0.05**
**APTT (seconds)**	Day 0	55	36.9 (35.4, 39.8)	55	38.6 (36.3, 42.9)	0.20
**(31.5–43.5)**	Day 3	45	37.7 (35.2, 39.3)	50	40.1 (36.8, 44.2)	**0.02**
	Day 7	58	36.9 (34.9, 38.9)	52	40.8 (36.5, 43.5)	**0.02**
**LDH (U/L)**	Day 0	55	203 (189, 240)	55	203 (178, 234)	0.43
**(120–250)**	Day 3	45	210 (176.5, 236.5)	53	199 (172, 226)	0.95
	Day 7	52	207 (179.3, 237.3)	52	200 (172.5, 246.8)	0.19
**TB (μmol/L)**	Day 0	55	11.3 (9.4, 14.8)	55	7.3 (6, 10.5)	<0.001
**(3.4–20.5)**	Day 3	45	8.8 (7.3, 11.7)	53	9.5 (7.2, 11.6)	0.64
	Day 7	53	9.3 (7.4, 11.7)	52	8.1 (7.2, 11.3)	0.67
**ALT(U/L)**	Day 0	55	29 (16, 45)	55	22 (13, 33)	0.30
**(8–38)**	Day 3	45	25 (15.5, 39)	53	19 (11, 28)	0.36
	Day 7	53	30 (18.5, 51.5)	52	20 (11.5, 34.5)	0.08
**CK (U/L)**	Day 0	55	89 (54, 126)	55	86 (56, 135)	0.42
(**30–200U/L**)	Day 3	39	60 (39, 85)	46	61.5 (41.5, 99.3)	0.42
	Day 7	39	60 (40, 80)	51	56 (40, 87)	0.19
**cTNI (ng/ml)**	Day 0	55	0.02 (0.02, 0.02)	55	0.02 (0.01, 0.04)	0.28
**(<0.04)**	Day 3	35	0.02 (0.02, 0.02)	50	0.02 (0.01, 0.03)	0.26
	Day 7	39	0.02 (0.02, 0.02)	49	0.02 (0.01, 0.02)	0.48
**Pre-albumin (mg/L)**	Day 0	55	195.2 (156.9, 256.1)	55	206 (137.7, 248.6)	0.35
**(180–400)**	Day 3	45	194.9 (152.7, 241.3)	52	199.2 (132.4, 261.7)	0.99
	Day 7	51	257.5 (215.5, 295.7)	52	236.9 (157.8, 284.4)	0.09

HDIVC, high dose intravenous vitamin C; DD, D-Dimer; APTT, activated partial thromboplastin time LDH, lactate dehydrogenase (LDH); TB, total bilirubin; ALT, alanine transaminase (ALT); CK, creatine kinase (CK); cTNI, cardiac troponin I; RR, relative risk; CI, confidential interval; IQR, interquartile range. P, HDIVC vs control group.

**FIGURE 3:**
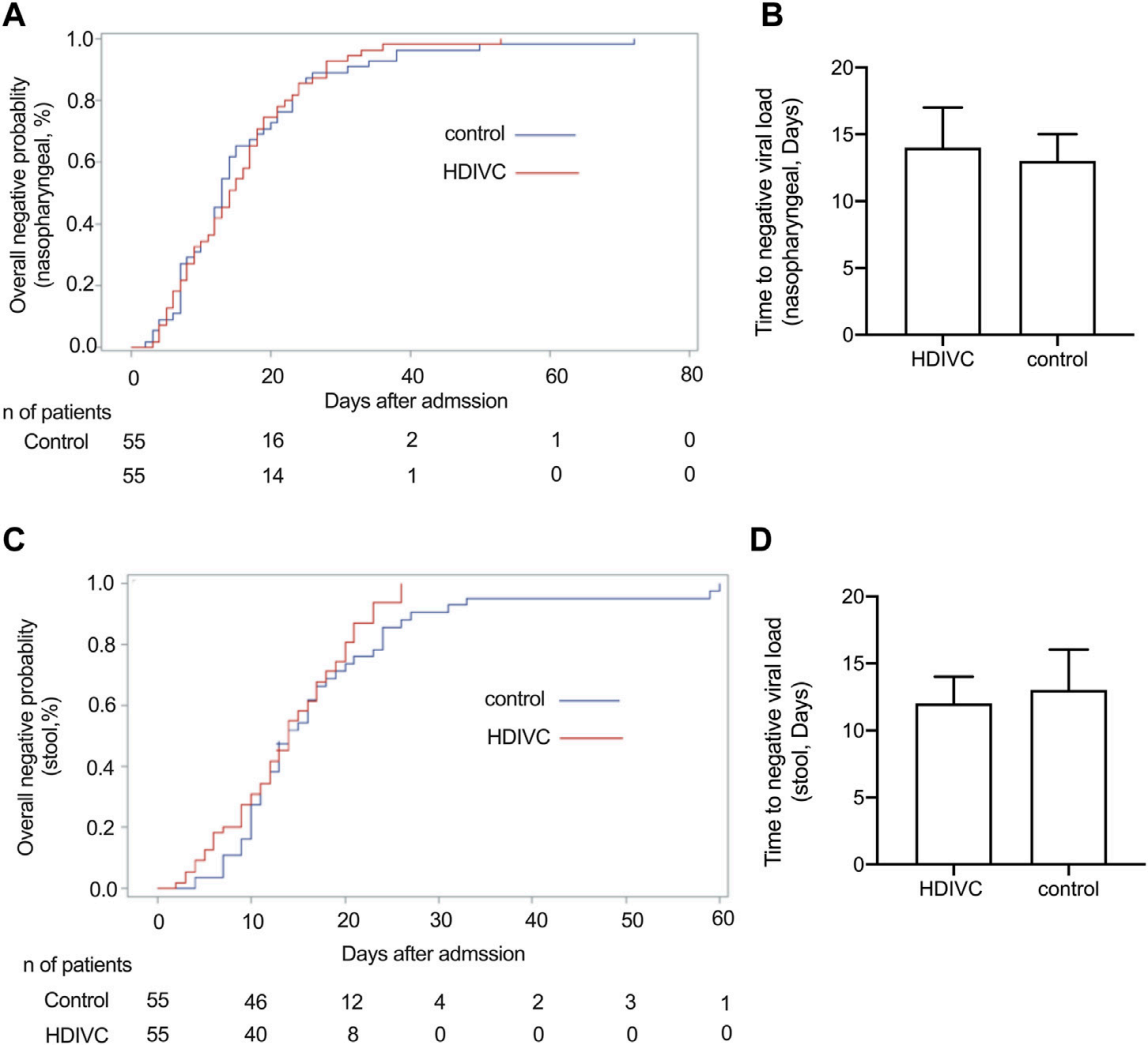
Effect of HDIVC on the time to negative nucleic acid load. The overall negative probability of nasopharyngeal swab **(A)** and stool **(C)** at admission between the HDIVC and control groups were compared and no significant difference was found. The time to negative nucleic acid was compared between HDIVC and control groups for nasopharyngeal swab **(B)**, median [IQR], days, 14 [8, 21] vs 13 [7, 21], *P* = 0.79) and for stool **(D)**, median [IQR], days, 12 [7, 17] vs 13 [10, 20], *P* = 0.12). HDIVC, high dose intravenous vitamin C; COVID-19, coronavirus disease 2019. IQR, interquartile range.

No significant difference in the time to achieve negative viral load of nasopharyngeal swab (Figures 3A,B) and stool (Figures 3C,**D**) was observed between the HDIVC group and the control group.

## Discussion

In this retrospective study, we found that after application of the HIDVC protocol since March 23, 2020, fewer (4/55 vs 12/55, RR = 0.28 [0.08, 0.93], *P* = 0.03) patients with moderate COVID-19 on admission evolved to the severe type during the week after admission. These patients also demonstrated a shorter SIRS duration and a lower CRP level. The patients with CD4^+^ T cell deficiency on admission who accepted HDIVC showed a better recovery ability of the CD4^+^ T cell count than those who had not received HDIVC. Coagulation function indicators, including APTT and D-dimer, were improved in the HDIVC group compared to the control group.

According to the recent report (Chiscano-Camón et al., 2020), the level of vitamin C is almost undetectable in the COVID-19 patients with severe or critical condition. Another recent study also reported low vitamin C plasma levels in COVID-19 patients, and non-survivors had half the plasma level of survivors (Arvinte et al., 2020). Therefore, early application of HIDVC may assist the quick recovery of its level and gain the benefits as we observed. We found obvious differences in the primary outcome, the disease aggravation, between the two groups. This finding implies the effect of HDIVC in the prevention of disease aggravation. This was partially consistent with the mortality reducing effect of HDIVC on sepsis with acute respiratory distress syndrome reported by Folwer (CITRIS-ALI study) (Fowler et al., 2019) and Hemilä, et al. (reanalysis of CITRIS-ALI study) (Hemilä and Chalker, 2020).

Recently, Zhang et al. (2021) reported that HDIVC (12 g every 12 h, 7 days) failed to improve invasive mechanical ventilation-free days in 28 days (the primary outcome). Compared to our study, the patients enrolled in their study were with higher severity of disease and the duration from onset of symptom to administration of HDIVC (median [IQR], 17 [11–25], days) of their study was longer than ours (control group: 3 [2–7], HDIVC group: 4 [2–6], days). Therefore, it is speculated that the early application of HDIVC routinely in COVID-19, especially when there is a potential risk of disease aggravation, may gain benefits. It should be noted that our study design was a comparison between two groups of patients before and after HDIVC protocol initiation. We matched the two groups strictly and the other therapy showed no significant difference, but as the understanding and management of COVID-19 improves, the outcomes may be better during the time of HDIVC administration than in the previous two months. Therefore, high quality randomized controlled trials are warranted for the prevention of disease aggravation using HDIVC.

SIRS, characterized by the release of huge amounts of pro-inflammatory cytokines, including tumor necrosis factor-α, interleukin-1β, interleukin-6, and interferon-γ named as “cytokine storm”, has been reported to be correlated with higher mortality in severe sepsis (Kaukonen et al., 2015). The Cytokine storm is regarded as an important characteristic in the early stages of COVID-19 (Fink-Neuboeck et al., 2016). The relevance of the cytokine storm to COVID-19 is still in debate and several clinical trials are underway (NCT04306705, NCT04322773) to investigate its potential role as a therapeutic target (Sinha et al., 2020). Although we did not directly show the effect of HDIVC on cytokines, we have demonstrated the shorter duration of SIRS and less SIRS prevalence in the HDIVC compared to the control group during the first week after admission. Serum levels of CRP are usually used to track and monitor the inflammatory response caused by infection due its short half-life of 19 h (Williams et al., 2019). CRP levels were shown to be reduced rapidly by HDIVC (200 mg/kg/day) in a previous before-after study in a cohort of sepsis patients (Fowler et al., 2014). In this study, we found that CRP levels in the HDIVC group were significantly lower than the ones in the control group. Therefore, we concluded that HDIVC might be beneficial for the inhibition of the inflammatory response in COVID-19 patients.

A reduction of lymphocytes, especially in the CD4^+^ T cell subgroup, has been reported to correlate with COVID-19 severity (Xu et al., 2020). SARS-CoV-2 infects and kills T lymphocyte cells. This might be due to growth inhibition and apoptosis of hematopoietic cells by the production of autoimmune antibodies (Yang et al., 2004) or certain cytokines (Channappanavar et al., 2014). In our study, 12 out of 55 patients in the HDIVC group and 18 out of 55 patients in the control group had CD4^+^ T cell deficiency on admission. Lymphocytes, especially T lymphocytes, have been extensively studied in the context of vitamin C biology (van Gorkom et al., 2018). Both *in vitro* and *in vivo* studies have shown that vitamin C is essential for the development, maturation, and proliferation of functional T lymphocytes, and epigenetic regulation of gene expression is one of the underlying mechanisms (Manning et al., 2013). We showed among the patients with CD4^+^ T cell deficiency on admission, the increase in CD4^+^ was more obvious in the HDIVC group than in the control group. This finding might imply the immune-enhancing property of HDIVC in the treatment of COVID-19.

Coagulopathy is a common feature of SARS-CoV-2 infection, and an increase in D-dimer level is the most common finding (Iba et al., 2020a), occurring in 43% of non-severe case (Guan et al., 2020). Higher D-dimer and fibrin degradation product levels, longer prothrombin time, and longer APTT have been reported to correlate with disease severity (Cheng, 2020). In our study, the APTT and D-dimer values were also in the normal range on admission, and we found that the APTT was shorter in the HDIVC than in the control group on Day 3 as well as Day 7, and the level of D-Dimer was lower in the HDIVC group than in the control group on Day 7. This confirmed the beneficial effect of HDIVC on coagulation disorders. This finding might be explained by the fact that vitamin C exerts an improving effect on endothelial damage (Barabutis et al., 2017), which promotes microvascular clot formation and angiopathy in COVID-19 pneumonia (Iba et al., 2020b).

## Conclusion

In this retrospective before-after study, we found that fewer COVID-19 pneumonia patients suffered disease aggravation after HDIVC application. Significant differences in the duration of SIRS, CRP level, CD4^+^ T cell recovery, and coagulation function indicators were found between the HDIVC and control groups. These results imply that HDIVC may have a role in prevention of the disease aggravation, possibly due to its improvement of the inflammatory response, immune function and coagulation function. Anyway, these observations require evaluation in prospective clinical trials.

This study was conducted in accordance with the amended Declaration of Helsinki (as revised in 2013) and approved by the institutional ethics board of Ruijin Hospital, Shanghai Jiao Tong University School of Medicine. Oral consent was obtained from each participated patient.; Consent for publication; All the authors approved the publication.; Availability of supporting data; All data are fully available without restriction.; Competing interests; The authors declare that they have no competing interests.

## Data Availability

The original contributions presented in the study are included in the article/Supplementary Material, further inquiries can be directed to the corresponding authors.

## Abbreviations

HDIVC, high dose intravenous vitamin C; SARS-CoV-2, Severe acute respiratory syndrome coronavirus 2; COVID-19, coronavirus disease 2019; SIRS, systemic inflammatory response syndrome; CRP, C-reactive protein; APTT, activated partial thromboplastin time; ROS, reactive oxygenase species; ESR, erythrocyte sedimentation rate; LDH, lactate dehydrogenase (LDH); TB, total bilirubin; ALT, alanine transaminase; CK, creatine kinase; cTNI, cardiac troponin I; IQR, interquartile range; RR, relative risk; CI, confidential interval; Ethical Approval and Consent to participate.
