# Supplementary material for: High Dose Intravenous Vitamin C for Preventing The Disease Aggravation of Moderate COVID-19 Pneumonia. A Retrospective Propensity Matched Before-After Study
Source: Front Pharmacol. 2021 Apr 22;12:638556. doi: 10.3389/fphar.2021.638556 (PMC8100592; doi:10.3389/fphar.2021.638556)
Supplement: Supplementary file 1 [file table1.docx]

Supplementary Table 1 Secondary outcomes

SIRS: systemic inflammatory response syndrome; CRP: C-reactive protein; ESR: erythrocyte sedimentation rate; Day 0: the day on admission; Day 7: 6-7 days after admission; DD: D-Dimer; APTT: activated partial thromboplastin time LDH: lactate dehydrogenase (LDH); TB: total bilirubin; ALT: alanine transaminase (ALT); CK: creatine kinase (CK); cTNI: cardiac troponin I;

| **Secondary outcomes** | **Items** |
| --- | --- |
| **Inflammatory response** | 1) including the SIRS occurrence on Day 0 and Day 7;  2) the duration of SIRS within the first week after admission;  3) serum CRP level;  4) serum ESR level |
| **Immune function** | 1) the number of patients with deficiency of CD4+ T cell (< 410/ml), CD8+ T cell (< 190/ml) and lymphocyte (< 1.1 *10^9/L) |
|  | 2) the changes of CD4+ T cell, CD8+ T cell and lymphocyte of the patients with immune deficiency from Day 0 to Day 7 |
| **Organ function** | 1) coagulation function indicators: APTT and serum level of D-dimer  2) hepatic function indicators: serum level of LDH, ALT and TB;  3) cardiac function indicators: serum level of CK and cTNI  4) nutritional status indicator: serum level of pre-albumin |
| **Time to viral load negative** | 1) nasopharyngeal swab  2) stool |
