# Supplementary material for: High Dose Intravenous Vitamin C for Preventing The Disease Aggravation of Moderate COVID-19 Pneumonia. A Retrospective Propensity Matched Before-After Study
Source: Front Pharmacol. 2021 Apr 22;12:638556. doi: 10.3389/fphar.2021.638556 (PMC8100592; doi:10.3389/fphar.2021.638556)
Supplement: Supplementary file 2 [file table2.docx]

| **Variables**  **(median [IQR])** | **Time points** | **n** | **HDIVC** | **n** | **Control** | **P value** |
| --- | --- | --- | --- | --- | --- | --- |
| **CD4+ T cell, n/μl** | Day0 | 55 | 535 (415, 721) | 54 | 558 (368, 795.3) | 0.85 |
|  | Day3 | 39 | 675 (475, 866) | 21 | 731 (540, 918.5) | 0.57 |
|  | Day7 | 42 | 752 (640, 1008) | 28 | 720 (565.8, 1019.8) | 0.81 |
| **CD8+ T cell, n/μl** | Day0 | 55 | 375 (273, 498) | 54 | 349.5 (202.8, 566.3) | 0.97 |
|  | Day3 | 39 | 545 (313.3, 666) | 21 | 478.7 (291.5, 754.5) | 0.73 |
|  | Day7 | 42 | 543.5 (387.3, 687.5) | 28 | 471 (310.3, 739.3) | 0.34 |
| **Lymphocyte,** | Day0 | 55 | 1.4 (1.1, 1.7) | 55 | 1.3 (1.0, 1.9) | 0.92 |
| **n*10^9/l** | Day3 | 47 | 1.8 (1.4, 2.0) | 52 | 1.7 (1.2, 2.2) | 0.83 |
|  | Day7 | 55 | 1.9 (1.5, 2.3) | 52 | 1.9 (1.3, 2.3) | 0.43 |

**Supplemental Table 2 Effect of HDIVC on immune function.**

HDIVC: high dose intravenous vitamin C; Day0: the day on admission; Day3: 3-4 days after admission; Day7: 6-7 days after admission; IQR: interquartile range
